# Effect of electro-acupuncture on postpartum urinary retention: a protocol for multicenter, randomized and placebo-controlled trial

**DOI:** 10.3389/fmed.2025.1698938

**Published:** 2025-11-20

**Authors:** Zhen Dou, Yunzhi Zhang, Hongyan Cui, Xiaoli Zhao, Lijing Dai, Baojuan Wang, Yu Fu, Tian Xia, Ying Chang

**Affiliations:** 1 Department of Reproductive Medicine, First Teaching Hospital of Tianjin University of Traditional Chinese Medicine, National Clinical Research Center for Chinese Medicine Acupuncture and Moxibustion, Tianjin, China; 2 School of Traditional Chinese Medicine, Beijing University of Chinese Medicine, Beijing, China; 3 Department of Obstetrics, Tianjin Central Hospital of Gynecology Obstetrics, Tianjin, China; 4 Second People's Hospital of Fengrun District, Tangshan City, Tangshan, Hebei, China; 5 Department of Acupuncture, First Teaching Hospital of Tianjin University of Traditional Chinese Medicine, National Clinical Research Center for Chinese Medicine Acupuncture and Moxibustion, Tianjin, China; 6 Tianjin Central Hospital of Gynecology Obstetrics, Tianjin, China

**Keywords:** postpartum urinary retention, electroacupuncture, controlled clinical trial, bladder function recovery, non-pharmacological therapy

## Abstract

**Background:**

Postpartum urinary retention (PUR) is a common and clinically relevant complication after delivery. Although urinary catheterization can provide temporary relief from voiding disorders, it is associated with discomfort, pain, potential urethral trauma, and an increased risk of urinary tract infection. Preliminary randomized and observational studies have suggested that acupuncture may facilitate voiding and early bladder recovery in PUR; however, the current evidence base remains limited by small sample sizes, heterogeneous methods, and risk of bias. To address these gaps, we designed a multicenter, randomized, sham-controlled clinical trial to evaluate whether electroacupuncture (EA) reduces the incidence of PUR and improves early bladder recovery.

**Methods:**

This is a multicenter, randomized, parallel-group clinical trial conducted in China to investigate the impact of EA intervention on the incidence of PUR. Patient screening and enrollment will take place at four hospitals in China: the First Teaching Hospital of Tianjin University of Traditional Chinese Medicine, Tianjin Central Hospital of Gynecology Obstetrics, Tianjin Shuige Hospital and Second People’s Hospital of Fengrun District, Tangshan City. Patients will be randomly assigned to either the EA group or the sham acupuncture group, with 330 patients in each group. Each acupuncture treatment will comprise three 30-min sessions over 2 days (the first hour, the first day, and the second day after delivery). The primary outcome measure is the incidence of PUR. Adverse events will be recorded, and their impact will be analyzed at the end of the trial.

**Conclusion:**

This multicenter, randomized, sham-controlled trial is designed to rigorously evaluate whether EA reduces the incidence of PUR and accelerates early bladder recovery. The findings will contribute evidence to inform the role of non-pharmacologic interventions in postpartum rehabilitation.

**Clinical trial registration:**

Chinese Clinical Trial Registry, ChiCTR2300078039.

## 1 Introduction

Postpartum urinary retention (PUR) is a serious and common clinical complication after delivery (1), mainly manifested as voiding disorders or incomplete bladder emptying. Based on its different clinical manifestations, PUR can be classified into overt, covert, and persistent PUR. Overt PUR refers to the inability to void 6 or more hours after vaginal delivery or removal of catheter after cesarean section. Covert PUR is defined as post-void volume (PVR) of >150 mL measured by ultrasound or catheterization after spontaneous urination. Persistent PUR is urinary retention lasting beyond 3 days postpartum and requires indwelling catheter or intermittent self-catheterization (2, 3).

Based on differences in diagnostic criteria, measurement methods, and timing, the incidence of PUR varies from 1.5 to 45% (4). However, covert PUR is somewhat concealed, making clinical screening and management more challenging, and it is more prone to delayed diagnosis and misdiagnosis. Although some studies have shown that covert PUR can normalize within a few days, even a single over-distension of the bladder can cause long-term urination disturbance, affecting the long-term function of the bladder, leading to recurrent urinary tract infections, and even renal failure (5). Additionally, PUR can affect uterine involution, resulting in increased postpartum bleeding, which imposes a heavy psychological burden on the parturients and seriously impacts their physical and mental recovery, as well as their quality of life and long-term health (4). Therefore, early diagnosis and prevention of PUR are of great significance for the physical and mental well-being of parturients.

The clinical treatment of PUR mainly involves urethral catheterization, drug therapy, physical therapy, and pelvic floor muscle functional exercises (6). While urinary catheterization can temporarily alleviate the clinical symptoms of voiding disorders, it does not offer a complete cure. Additionally, this procedure can cause maternal discomfort, pain, and anxiety, and even urethral injury, increasing the risk of urinary system infections (7, 8). Studies have shown that urinary tract infections caused by urinary catheter account for approximately 60 to 80% of patients with urinary tract infections, and long-term indwelling urinary catheters can weaken bladder autonomic function, which is not conducive to recovery (8). Drug therapy, such as tamsulosin, neostigmine, and bethanechol, mainly acts on bladder and sympathetic nervous system receptors, and its impact on improving post-void residual bladder volume is unclear. Additionally, these drugs have side effects such as tachycardia, diarrhea, and vomiting, and it is unknown whether they are secreted in human milk, thus safety considerations for maternal breastfeeding are lacking (2). Conventional non-invasive methods, such as functional exercises and acupoint hot compresses, are easy to accept but have limited efficacy for patients with severe PUR or complications, and the long-term effect is unclear (8). Therefore, it is urgent to find a safe, effective, less side effect-prone, and long-term curative approach for the prevention and treatment of PUR.

Acupuncture is practiced internationally and, for selected indications, has been incorporated into some clinical practice guidelines; however, recommendations and the underlying evidence quality vary by condition (9). For urinary retention—particularly PUR—preliminary randomized and observational studies suggest that acupuncture, including EA, may facilitate spontaneous voiding and early bladder recovery (10). Yet existing data are constrained by small sample sizes, heterogeneous interventions and comparators, short follow-up, and risk of bias; high-quality multicenter, sham-controlled trials remain scarce, and important questions persist regarding covert PUR and longer-term outcomes. Taken together, these limitations necessitate a rigorously designed multicenter, randomized, sham-controlled trial to establish whether EA confers clinically meaningful benefits for PUR, including covert PUR and longer-term outcomes (11).

## 2 Methods

### 2.1 Study design

**Figure 1:**
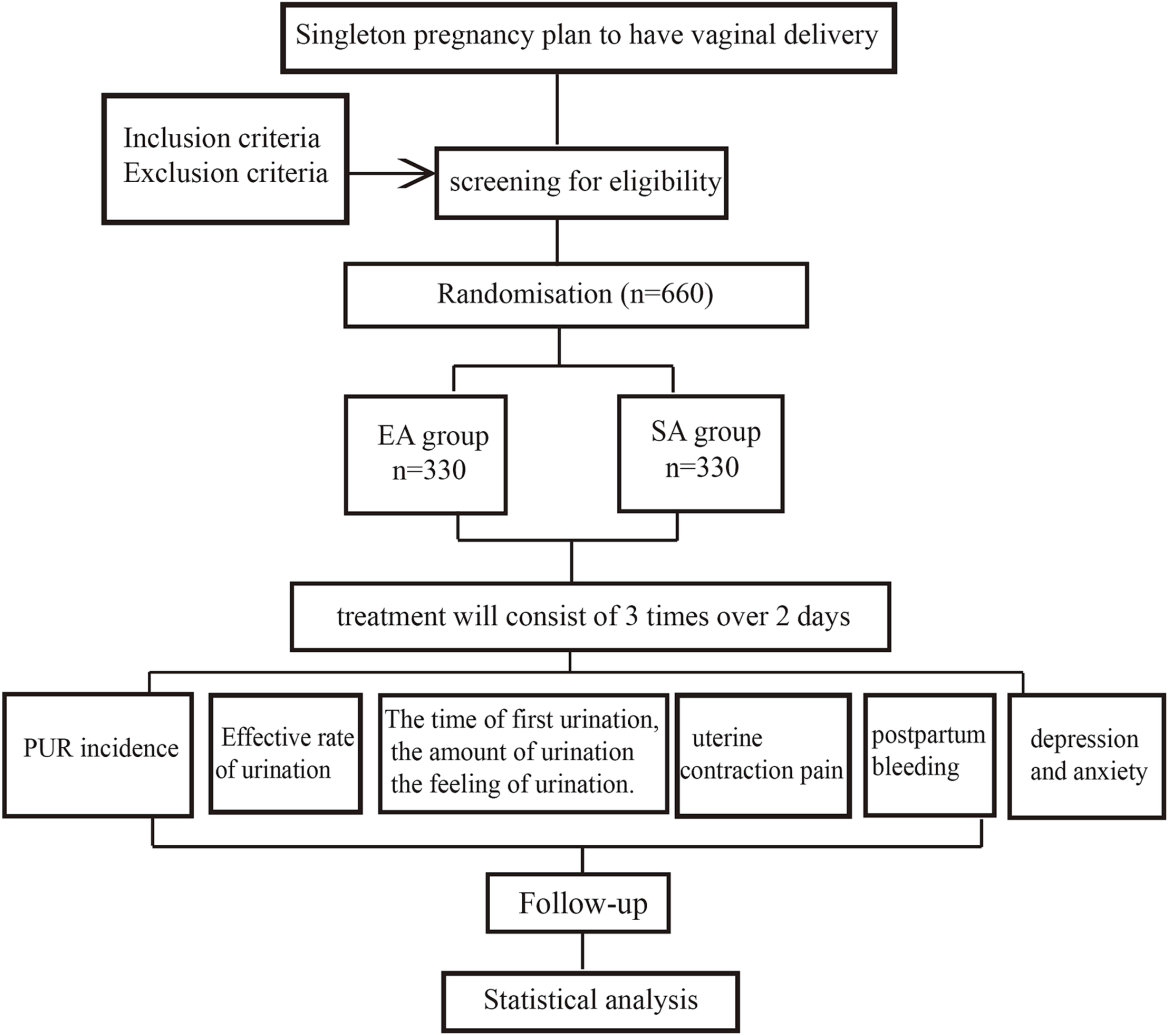
The study flow chart.

This is a multicenter, randomized, parallel-group clinical trial conducted in China to investigate the impact of EA intervention on the incidence of PUR. The study protocol is to prefer the Consolidated Standards of Reporting Trials (CONSORT) guidelines (12) and the Standards for Reporting Interventions in Clinical Trials of Acupuncture (STRICTA) (13), as well as with the Standard Protocol Items: Recommendations for Interventional Trials (SPIRIT) (14) statement the study will commence following ethics approval and registration protocol. Patient screening and inclusion will start in August 2024. It has been registered at Chinese Clinical Trials Registry (ChiCTR2300078039). Figure 1 is the study flow chart and Table 1 shows the schedule of enrolment, interventions and assessments.

**Table 1:** Schedule of trial enrolment, interventions and assessments.

Period	Enrolment	Allocation	Treatment			Assessment				Follow-up	
Time points	Before delivery		1 h	24 h	48 h	the 1st urination	6 h	24 h	48 h	6–8 weeks	1 year
Enrolment											
Eligibility screen	X										
Informed consent	X										
Randomization		X									
Intervention											
Electro-acupuncture			X	X	X						
Sham acupuncture			X	X	X						
Assessments											
Postpartum urinary retention						X	X	X	X		
The time of first urination						X					
The amount of first urination						X					
The feeling of unobstructed first urination (TCM syndrome quantification scale)						X				X	
Effective rate of urination						X					
Postpartum uterine contraction pain (VAS)							X	X	X		
The amount of 24 h postpartum bleeding								X			
Postpartum depression and anxiety (EPDS)									X		X
Pelvic floor dysfunction questionnaire (PFDI-20)											X
Blindling									X		
Adverse events								X	X		

### 2.2 Participants

#### 2.2.1 Recruitment and withdrawal

Patient screening and recruitment will take place at four hospitals in China: the First Teaching Hospital of Tianjin University of Traditional Chinese Medicine, Tianjin Central Hospital of Gynecology Obstetrics, Tianjin Shuige Hospital and Second People’s Hospital of Fengrun District, Tangshan City. The target enrollment is set at 660 patients. Obstetricians responsible for identifying eligible patients will be briefed about the study, and these patients will then be given the option to decide whether they want to participate. After signing the informed consent form, patients will be randomly assigned to either the electro-acupuncture group or the sham acupuncture group, with 330 patients allocated to each group. It is important to emphasize that patients have the right to withdraw from the study at any stage.

#### 2.2.2 Inclusion criteria

Patients meeting the following additional inclusion criteria may be included:

1. Primiparous women aged 20 to 45 years with singleton pregnancies planning for vaginal delivery.
2. Delivery at a gestational age of 37 to 42 weeks.
3. Being clearly conscious and cooperative.
4. Signing the informed consent.

#### 2.2.3 Exclusion criteria

Patients meeting any of the following conditions will be excluded:

1. Termination of pregnancy due to fetal malformation or stillbirth.
2. Severe central nervous system diseases, internal and surgical diseases, mental and psychological diseases, urogenital-related diseases, infectious diseases, and long-term drug treatment.
3. Severe coagulation disorders and a tendency towards spontaneous bleeding.
4. Skin damage at the acupuncture site.
5. Refusal to follow-up.
6. Refusal to sign the informed consent form.

#### 2.2.4 Drop-out and discontinuation criteria

All randomized participants will be included in the Intention-to-Treat (ITT) analysis. The Full Analysis Set (FAS) will be identical to the ITT population. Participants who meet any of the following protocol deviation criteria will be excluded from the Per-Protocol (PP) analysis but retained in the ITT/FAS. All participants who receive at least one session of acupuncture (active or sham) will be included in the Safety Analysis Set. Sensitivity analyses will be conducted to explore the impact of major protocol deviations.

1. Rescue measures are implemented due to postpartum hemorrhage, eclampsia, or other obstetric conditions;
2. Conversion from vaginal delivery to cesarean section.
3. Infection is diagnosed during delivery;
4. An indwelling catheter is used and diuretics are administered due to other diseases within 6 h after delivery;
5. A degree III laceration above the perineal fissure is diagnosed;
6. Due to other postpartum reasons, the number of treatment sessions received by patients is less than 3 times, which affects the analysis and judgment of the results;
7. The researchers can terminate the patients from the study at any stage if any events occur that may affect the safety of patients in the study.

### 2.3 Randomization and blinding

This study will use the network-based clinical trial public management platform (ResMan, http://www.medresman.org.cn/login.aspx) to implement centralized randomization. Randomization will be carried out using stratified block randomization (with block sizes of 4 or 6) according to the four participating research centers, and allocation concealment will be achieved through the central randomization system of the ResMan platform. After patients complete baseline assessments and are confirmed to be eligible, independent researchers will log into the system to obtain an unpredictable, automatically generated random allocation sequence. Group allocation information will be blinded to the study participants, outcome assessors (including personnel who perform BVI-9400 bladder scans, EPDS scoring, and other questionnaire assessments), and data analysts. To maintain blinding consistently across all study centers, these assessors will not be involved in any treatment-related activities and will receive standardized training on blinded assessment procedures. Furthermore, data entry will be performed by independent personnel who are not involved in patient recruitment or treatment delivery. The sham procedure uses non-penetrating needles and 0 mA current to mimic active treatment without physiological effects. At the end of the trial, the James Blinding Index will be used to quantitatively analyze the success degree of blinding. However, due to operational requirements, acupuncture therapists cannot be blinded.

After the final acupuncture treatment, all patients will be asked, “Do you believe that acupuncture has penetrated the skin?” The response will be categorized into three options:

- Acupuncture penetrated the skin;
- Acupuncture did not penetrate the skin;
- I do not know.

This will be taken into consideration to evaluate the success of blinding.

### 2.4 Interventions

Each acupuncture treatment modality will involve three sessions, each lasting 30 min, administered over two consecutive days (first hour, first day, second day after delivery). This intensive regimen (three sessions within 48 h postpartum) was chosen because the initial 6–12 h postpartum represent the critical window for the onset of PUR. The goal is to provide repeated neuromodulatory stimulation during this period to maximize preventive effects on bladder recovery. The selection of acupuncture points is based on traditional Chinese medicine theory, evidence-based clinical research, and our accumulated clinical experience. The treatment protocol adheres to CONSORT and STRICTA recommendations, providing comprehensive details on the treatment, including the number of needles used, session frequency, and treatment duration. Only licensed acupuncturists holding a master’s degree and possessing more than 2 years of acupuncture experience will conduct the treatment. Prior to the study commencement, all acupuncturists at each sub-center will receive instruction in the theory of PUR and standardized operating procedures.

#### 2.4.1 Electro-acupuncture group

**Figure 2:**
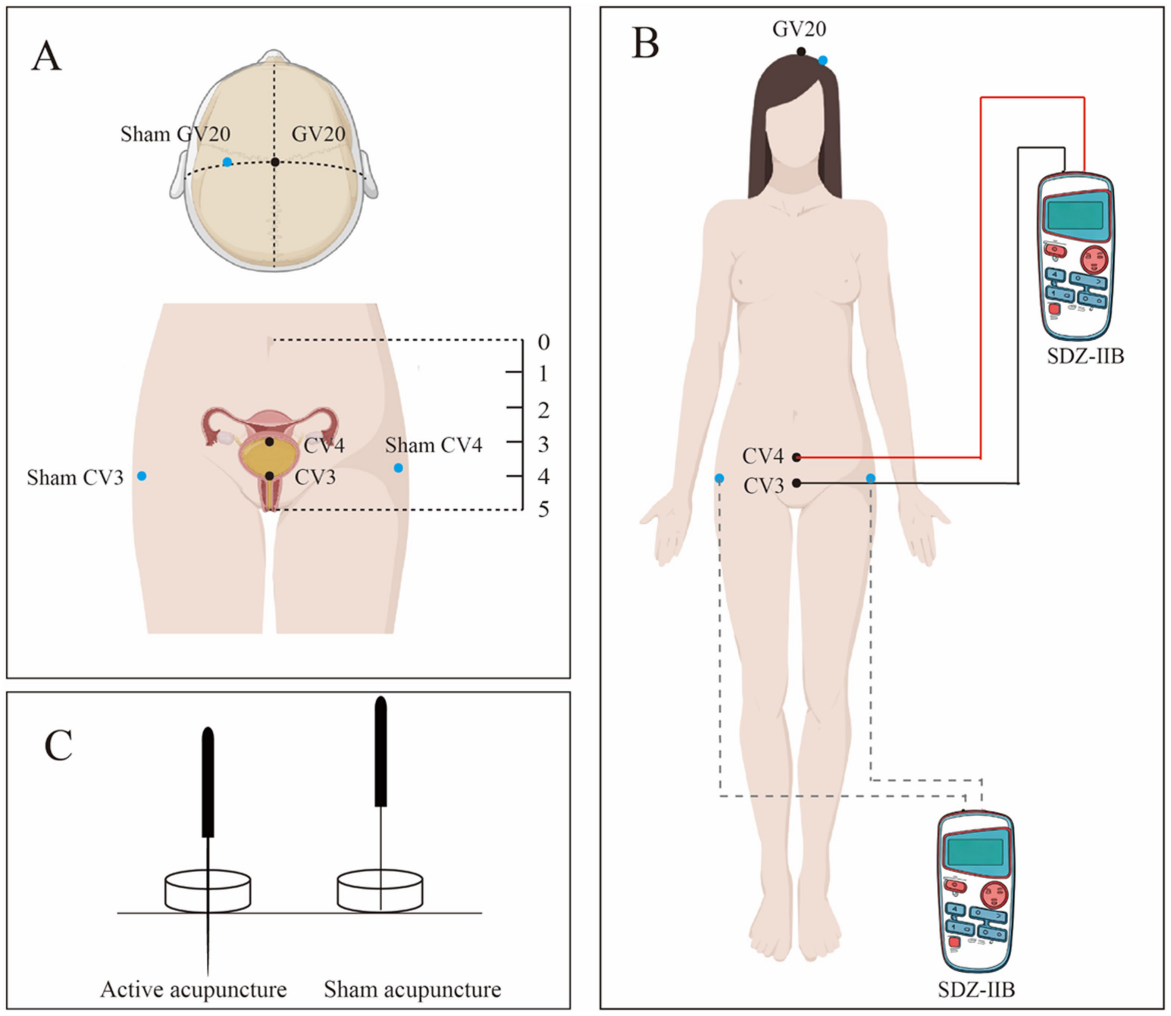
**(A)** Shows the specific location of the two groups of acupoints; **(B)** Shows the connection method of the EA apparatus; **(C)** Shows active acupuncture and sham acupuncture.

The selected acupoints will include Baihui (GV20), Zhongji (CV3), and Guanyuan (CV4), adhering strictly to the National Acupoint Standard of the People’s Republic of China in 2021 (GB/T 12346–2021). The first treatment session will be initiated within 1 h postpartum (±30 min). Detailed positioning is illustrated in Table 2 and Figure 2. Patients will be positioned supine during the procedure. Adhesive pads will be applied to the skin over the acupoints, followed by the insertion of single-use acupuncture needles (0.25 mm * 40 mm, Hwato, Suzhou, China) through the adhesive pads.

**Table 2:** Locations and needling methods of acupoints for electro-acupuncture group.

Acupoint	Location	Needling method
Baihui (GV20)	On the head, 5 inches above the center of the front hairline.	Depth of 10-15 mm and an angle parallel to the scalp, then adopt the method of small amplitude and high frequency twisting(the range of twirling of the needle was 90°- 180°, and the frequency was 100 to 120 times a minute) for 1 min.
Zhongji (CV3)	In the lower abdomen, 4 cun below the umbilicus, on the anterior median line.	Obliquely acupunctured 10-20 mm and at a 45° angle to the abdomen.
Guanyuan (CV4)	In the lower abdomen, 3 cun below the umbilicus, on the anterior median line.	Obliquely acupunctured 10-20 mm and at a 45° angle to the abdomen.

The sequence and technique of acupuncture are as follows: GV20 will be needled at a depth of 10–15 mm with an angle parallel to the scalp, followed by small-amplitude, high-frequency twirling (90°–180°, 100–120 rotations/min for 1 min). CV3 and CV4 will then be obliquely punctured at 10–20 mm with a 45° abdominal angle and manipulated with the same twirling parameters for 1 min. Practitioners will apply standardized manual stimulation per STRICTA to elicit characteristic needling sensations (e.g., aching, heaviness, distension)—terms traditionally referred to as “de qi”; this terminology is used in its historical context and does not imply a validated biomedical mechanism. To operationalize sensation intensity, participants will rate needling sensations using a 0–10 Numeric Rating Scale (0 = no sensation, 10 = strongest imaginable sensation) immediately after needle manipulation, and practitioners will record perceived needle grasp (present/absent). Paired electrodes from an EA device (SDZ-IIB, Suzhou Medical Appliance) will be attached to the CV3 and CV4 needles, set to continuous 2 Hz, with current intensity adjusted individually between 1 and 5 mA to produce visible, comfortable local muscle twitching without pain. Needles will be retained for 30 min. The procedure and electrode connections are shown in Figure 2.

#### 2.4.2 Sham acupuncture group

Patients in the sham group will receive a non-insertive procedure matched to the EA group for time, attention, body position, and device appearance. Stimulation will be delivered at three predefined non-acupoint sites labeled sham-Baihui, sham-Zhongji, and sham-Guanyuan. Blunt-tip telescopic sham needles (0.25 mm× 40 mm, Hwato, Suzhou, China) will rest on the skin through adhesive guide tubes without skin penetration; no manual twirling will be performed and practitioners will not aim to elicit characteristic needling sensations (traditionally termed “de qi”). Leads from an EA device (SDZ-IIB, Suzhou Medical Appliance, Suzhou, China) will be attached to the sham-Zhongji and sham-Guanyuan simulators with the device powered on but current set to 0 mA (no output). The simulators will remain in place for 30 min, with the number and schedule of sessions identical to the EA group. Specific procedures are summarized in Table 3 and Figure 2.

**Table 3:** Locations and needling methods of non-acupoints for Sham group.

Acupoint	Location	Needling method
Sham-Baihui	2 cun lateral from GV20	Sham needles will not penetrate the skin
Sham-Zhongji	6 cun lateral from CV4(left)	Sham needles will not penetrate the skin
Sham-Guanyuan	6 cun lateral from CV4(right)	Sham needles will not penetrate the skin

### 2.5 Outcome measurements

At the baseline, clinical and sociodemographic data will be collected from all patients, encompassing:

(1) Basic Information:

- Age;
- Gestational age at delivery;
- Height;
- Weight (Predelivery);
- Body Mass Index (Predelivery);
- Conception mode.

(2) Management of the Delivery Process:

- Duration of labor;
- Use of epidural anesthesia;
- Incidence of episiotomy and perineal lacerations;
- Instances of vacuum-assisted and instrumental delivery;
- Process of placental delivery;
- Administration of uterine contractile drugs.

(3) Newborn Condition:

- Weight;
- Length;
- Apgar score.

#### 2.5.1 Primary outcome

The primary outcome is the PUR incidence.

The incidence of postpartum urinary retention (PUR) will be evaluated in accordance with the following criteria, which are grounded in the diagnostic guidelines outlined in “Postpartum urinary retention: an expert review” published in the American Journal of Obstetrics and Gynecology.

Overt PUR: Inability to void spontaneously within 6 h postpartum or within 6 h after removal of the indwelling catheter following cesarean section.
Covert PUR: Post-void residual (PVR) urine volume ≥ 150 mL after spontaneous voiding.
Persistent PUR: Requirement for an indwelling catheter or intermittent catheterization by the 3rd day postpartum.

Assessment time points are: the first urination after delivery, 6 h after delivery, the initial urination on the first day, and the second day following acupuncture. Meeting any of the aforementioned criteria at any assessment point will be considered as a positive PUR case.

**Figure 3:**
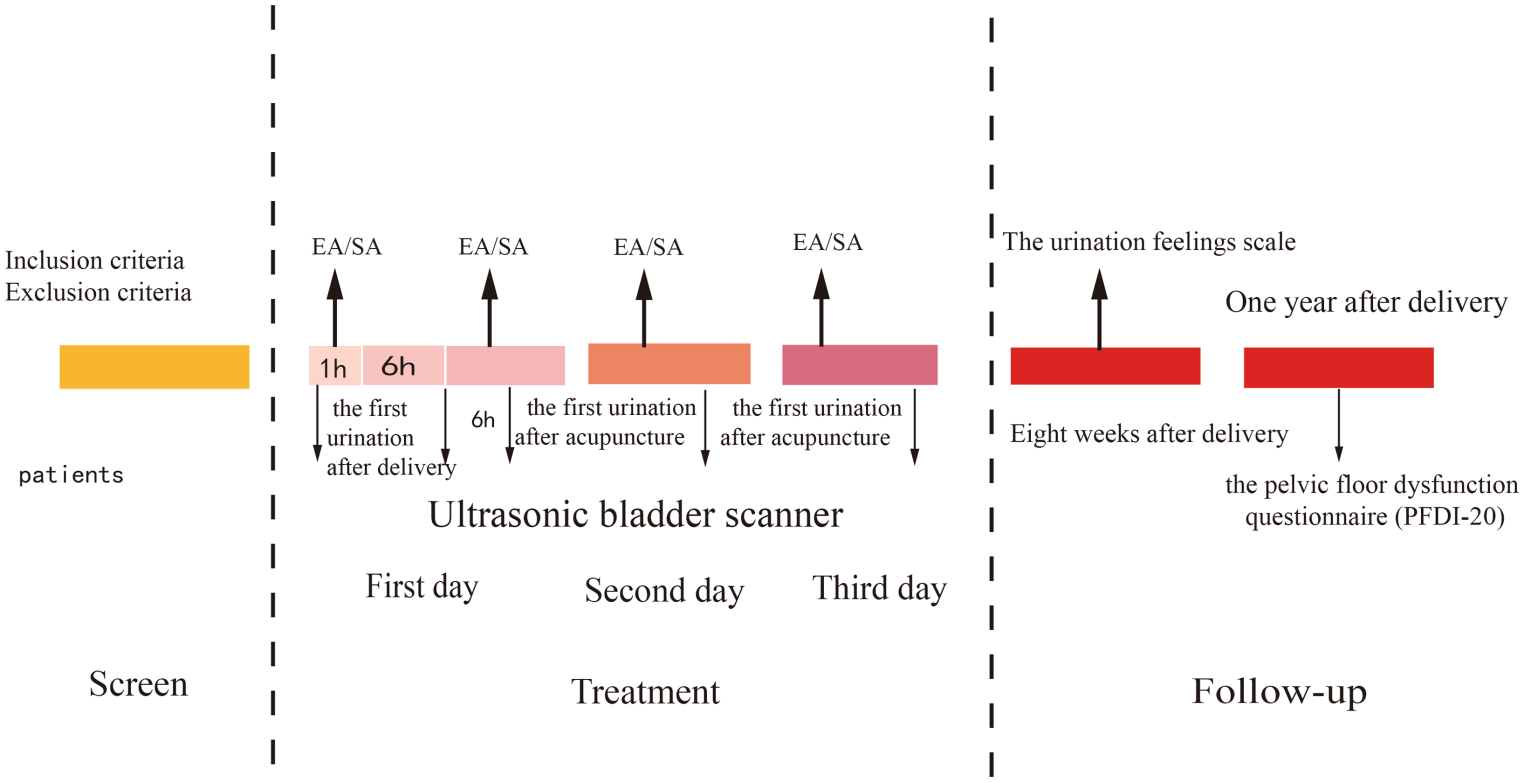
Intervention time nodes chart.

The PVR of the bladder will be measured using an Ultrasonic Bladder Scanner (BVI-9400 BladderScan^®^, Verathon Medical Europe, Ijsselstein, The Netherlands). All BladderScan BVI-9400 devices will be calibrated annually according to the manufacturer’s guidelines. Operators must complete a standardized training program and pass a competency assessment before performing study measurements. To ensure accuracy, any PVR measurement ≥150 mL will be immediately rechecked by a second, blinded operator within 15 min. The average of the two readings will be recorded as the final PVR value. The recorded PVR of the bladder and whether catheterization was required will be documented. The specific measurement times are illustrated in Figure 3.

#### 2.5.2 Secondary outcome

##### 2.5.2.1 The time of first urination, the amount of urination and the feeling of unobstructed urination

(1) Recording time from delivery completion to first urination:

The elapsed time from the conclusion of delivery to the initiation of the first urination will be recorded, measured in minutes (“min”).

(2) Recording volume of first post-delivery urination:

After urination in a urinal, the volume of urine will be measured using a cup, expressed in milliliters (“mL”).

(3) Assessment of unobstructed feeling during first urination:

The unobstructed sensation during the initial urination will be evaluated using the Traditional Chinese Medicine Syndrome Quantification Scale, where:

- 0 points: Unable to urinate;
- 2 points: Dribbling out urine, with a sense of difficulty;
- 4 points: Urination is acceptable, with a residual feeling;
- 6 points: Very smooth, without any residual sensation.

##### 2.5.2.2 Effective rate of urination

The classification of the first urination volume is as follows:

- A volume greater than 500 mL is considered significant;
- A volume ranging from 100 to 500 mL is deemed effective;
- A volume less than 100 mL is categorized as invalid.

The effective rate of urination is calculated using the formula: Effective rate of urination = (number of significant cases + number of effective cases)/total cases × 100%.

##### 2.5.2.3 Postpartum uterine contraction pain

The Visual Analogue Scale (VAS) will be employed to assess postpartum uterine contraction pain. The visual scale spans 10 centimeters, ranging from 0 to 10, where “0” represents a painless state, and “10” signifies the most unbearable pain. Scores will be assigned at specific time points, including 6 h after delivery, 2 h after acupuncture on the first day, and 2 h after acupuncture on the second day.

##### 2.5.2.4 Cumulative amount of 24 h postpartum bleeding

The volume of vaginal bleeding within the first 24 h after delivery will be documented using postpartum measuring pads. These pads will be changed regularly every 2–4 h, and upon replacement, they will be weighed and measured, with the unit of measurement being milliliters (“mL”). Blood loss will be measured using pre-weighed pads changed every 2–4 h, with volume (mL) calculated as weight difference (g) × 1.06 (density conversion factor). Permitted co-interventions include uterotonics (e.g., oxytocin) but exclude prophylactic catheterization. All co-interventions will be recorded and adjusted for in the analyses.

##### 2.5.2.5 Postpartum depression and anxiety

The Edinburgh Postpartum Depression Scale (EPDS) will be employed to assess depressive symptoms. The scale comprises 10 items, which will be administered after the final acupuncture session and again 6–8 weeks after delivery. Each item is graded on a four-point scale (0 to 3 points). A cumulative total score of ≥13 points indicates a diagnosis of postpartum depression.

#### 2.5.3 Safety assessment

Common adverse reactions associated with acupuncture include incidents such as broken needles, localized hematoma, bleeding, infection, and localized severe sharp pain. Post-delivery, common adverse reactions encompass late postpartum hemorrhage, postpartum infection, and constipation. Any adverse events (AEs) occurring during the study period will be thoroughly assessed, treated, and documented in the case report form (CRF).

### 2.6 Follow-up

#### 2.6.1 Follow-up 1—6–8 weeks after delivery

Patients will undergo follow-up through outpatient visits. Urination sensations will be assessed using the Traditional Chinese Medicine Syndrome Quantification Scale, where:

- 0 points: Unable to urinate;
- 2 points: Dribbling out urine, with a sense of difficulty;
- 4 points: Urination is acceptable, with a residual feeling;
- 6 points: Very smooth, without any residual sensation.

#### 2.6.2 Follow-up 2—1 year after delivery

Patients will be followed up via telephone. The assessment will include monitoring symptoms related to urination, defecation, and pelvic organ prolapse. Additionally, the Pelvic Floor Dysfunction Questionnaire (PFDI-20) will be administered.

We aim for ≥90% follow-up retention. The 6–8 week visit allows a ± 7-day window; the 1-year telephone follow-up allows ±30 days. Validated Chinese versions of EPDS and PFDI-20 will be administered by trained assessors.

### 2.7 Data management and monitoring

Case Report Forms (CRFs) will be developed to document and store individual patient data. To streamline data management and process monitoring, an electronic CRF system based on the ResMan platform will be implemented. Following data entry and resolution of any queries by researchers to ensure accuracy, database locking will be performed by data managers. All hardcopy and electronic study documents will be archived for a minimum of 5 years after publication. Readers or reviewers with data inquiries may contact the corresponding author to request access to the original data.

An independent Data and Safety Monitoring Board (DSMB, composition detailed in Table 4) has been established to safeguard participant safety and ensure data integrity. Comprising independent experts free from financial conflicts of interest, the DSMB will:

1. Conduct regular data reviews at six-month intervals;
2. Perform an interim analysis when 50% enrollment (*n* = 330) is reached, focusing on cumulative safety data (adverse events) and conducting futility assessments;
3. Provide confidential recommendations to the Trial Steering Committee regarding trial continuation, modification, or termination based on interim analysis findings.

**Table 4:** Data and Safety Monitoring Board (DSMB).

Member name	Affiliation	Roles and responsibilities
Linling Wu	First Teaching Hospital of Tianjin University of TCM	Review the protocol with respect to ethical and safety standards. Review the progress of the trial.
Ning Xue	First Teaching Hospital of Tianjin University of TCM	Monitor the safety of the trials. Review and interpret the data generated from the study.
Zhimei Zhao	First Teaching Hospital of Tianjin University of TCM	Adjudicate adverse events.

Prespecified trial stopping rules include: occurrence of ≥3 intervention-related serious adverse events (SAEs), or interim analysis indicating significant harm. All adverse events (AEs) and serious adverse events (SAEs) must be reported to both the DSMB and ethics committee within 24 h of documentation.

### 2.8 Quality control

To ensure the trials’ quality, standardized training will be provided to all acupuncturists, obstetricians, evaluators, and statisticians before the official launch of the clinical trial. The training will encompass research goals and content, patient recruitment, treatment procedures, patient communication skills, and outcome evaluation, aiming to maintain consistency across various patients and researchers. This approach ensures the feasibility and safety of the clinical research. Throughout the clinical trial, independent monitors will regularly visit each center to verify strict adherence to the research plan. They will also scrutinize the original data to ensure the CRFs are accurate, complete, and correct.

### 2.9 Sample size

This study employs a superiority design to evaluate the effect of electroacupuncture on the overall incidence of postpartum urinary retention (PUR), encompassing all subtypes (overt, covert, and persistent). Based on the research findings, the incidence of overt PUR in the intervention group is projected at 4.0%, while in the control group, it is estimated to be 9.0% based on previous observational data and a recent clinical trial in a similar population (4). The non-inferiority margin (*δ*) was set at 5%. Assuming a 1:1 ratio between the intervention and control groups, and utilizing a superiority test with a significance level (*α*) of 0.05, a power (1-*β*) of 80%, and accounting for a potential dropout rate of 10%, the study is planned to enroll 660 patients, evenly distributed with 330 in each group. The calculation formula is as follows:

$$n1=n2=[p_{1}(1-p_{1})+p_{2}(1-p_{2})]{\left(\frac{z_{1-\alpha }+z_{1-\beta }}{p_{1}-p_{2}-\delta }\right)}^{2}$$

While acknowledging that the initial effect size estimation was primarily informed by overt PUR data, we have implemented a comprehensive data collection system to capture all PUR subtypes, ensuring the primary outcome measure “PUR incidence” accurately reflects clinical reality. We anticipate this sample size will provide sufficient statistical power to detect clinically meaningful differences in overall PUR incidence across all subtypes. During final analysis, we will calibrate the effect size according to the actual observed incidence rates of all PUR subtypes, thereby ensuring proper alignment between the effect size and primary endpoint while enhancing the robustness and generalizability of our findings.

### 2.10 Statistical analysis

Demographic characteristics, general situations, and baseline conditions of the two groups will be compared and analyzed. For measurement data, an independent samples t-test or Wilcoxon rank-sum test will be employed, while χ^2^ test or Fisher test will be used for categorical data.

The incidence of PUR will be accurately assessed using Fisher’s exact test, and the relative risk (RR) along with the corresponding 95% confidence interval will be calculated. For other ordinal data, the Wilcoxon rank-sum test will be applied, while clinical counting data will undergo testing through the χ2 test. Postpartum depression and anxiety will be compared using Fisher’s exact test, and the RR along with the corresponding 95% confidence interval will be computed. Subject blinding effectiveness will be analyzed using the James Blinding Index. All statistical tests will be two-sided, and a *p* value less than 0.05 will be considered statistically significant for the tested differences.

For exploratory subgroup analysis, participants will be stratified into different subgroups based on age (< 40 years, ≥ 40 years), BMI (< 25 kg/m^2^, ≥ 25 kg/m^2^), duration of labor (< 700 min, ≥ 700 min), epidural anesthesia (with or without), vaginal midwifery techniques (forceps midwifery, fetal head attraction midwifery, fetal head rotation, perineotomy), gestational age (> 280 days, ≤ 280 days), and newborn birth weight (< 4,000 g, ≥ 4,000 g). The incidence difference between the two groups will be compared within each subgroup using the same statistical methods as mentioned above.

The primary analysis will follow the intention-to-treat (ITT) principle. Missing data will be handled using multiple imputation. A logistic regression model adjusting for study center will be used for the primary outcome. Secondary and subgroup analyses will apply Bonferroni correction for multiple comparisons.

GraphPad Prism 10.2 will be used for all statistical analysis.

## 3 Discussion

Urination is a complex process involving the coordinated actions of the cerebral cortex, spinal cord micturition center, peripheral nerves, and the lower urinary tract musculature (7). Disruptions to the muscle or nerve functions controlling urination during childbirth can impact normal micturition function, resulting in PUR (15). Preliminary clinical studies have explored acupuncture as a potential intervention for PUR, suggesting possible modulation of neurotransmitter pathways and improvements in urodynamic measures (16, 17). Preclinical work further reports that acupuncture may inhibit neuronal apoptosis, promote neurotrophic factor synthesis and secretion, support nerve repair, and thereby improve bladder function (18–20). In animal models, EA has been reported to activate afferent and efferent pelvic pathways, enhance detrusor contractility, and improve voiding-related outcomes (21). These mechanistic observations require confirmation in rigorously designed clinical studies.

In this trial, acupuncture is administered at three acupoints—GV20, CV3, and CV4—selected on the basis of traditional Chinese medicine practice and prior clinical use. Neuroanatomical hypotheses posit that the paracentral lobule contributes to voluntary micturition; its scalp projection approximates the vertex midline near GV20, and stimulation at GV20 may influence central pathways involved in voiding (22). Accordingly, needling GV20 may influence central pathways relevant to voiding. The urination activity of the bladder is regulated by lumbosacral circuits; parasympathetic (S2–S4) and sympathetic (T11–L2) outflow innervate the detrusor and internal sphincter. CV3 and CV4, located in the lower abdomen, receive segmental innervation primarily from T12–L4. Needling CV3/CV4 may modulate pelvic afferent–efferent signaling and support coordination of the sphincter and detrusor (23, 24). These mechanistic links remain hypothetical and are derived largely from physiological and preclinical observations. Accordingly, the present multicenter, randomized, sham-controlled trial will test whether electroacupuncture at GV20/CV3/CV4 facilitates spontaneous voiding and reduces post-void residual in PUR, while prospectively monitoring safety outcomes (25, 26)

Clinical research on acupuncture for PUR remains scarce internationally. A small clinical study suggested that acupuncture may alleviate urinary retention and facilitate catheter removal in some postpartum patients, potentially reducing exposure to indwelling-catheter discomfort and infection risk; however, the evidence is limited by nonrandom allocation, small sample size, single-center design, and short follow-up, resulting in low certainty (11). However, the study cited faces limitations such as a small sample size and the absence of random grouping, resulting in a low level of evidence. Urgent challenges persist in existing clinical research on acupuncture for PUR, including: 1. Lack of clarity in PUR diagnosis, with many studies focusing predominantly on overt PUR while overlooking the diagnosis and treatment of covert PUR, thereby posing potential risks to both short-term and long-term bladder function. The current evidence level for acupuncture treatment of PUR is suboptimal, and a dearth of multi-center, large-sample, randomized controlled clinical trials hampers the availability of high-quality data to support its efficacy.

To address the existing challenges in current clinical research, we have formulated a comprehensive strategy involving a multi-center, large-sample, randomized controlled clinical trial. This trial aims to assess the impact of EA intervention on both the incidence of PUR and the recovery of bladder function. Our investigation will encompass the three distinct categories of PUR, namely overt, covert, and persistent types. Additionally, we will explore the influence of EA on common postpartum conditions such as uterine contraction pain, postpartum bleeding, depression, and anxiety. A critical aspect of the study involves long-term observations through follow-up, where we will scrutinize the enduring effects of acupuncture on urination and pelvic floor function in postpartum individuals.

However, certain limitations are acknowledged in our study design. The nature of acupuncture treatment poses challenges to achieving practitioner blinding. Simultaneously, the administration of sham acupuncture may induce psychological effects on patients. The standardization of acupuncture points, applied uniformly across all subjects without syndrome differentiation, is another study limitation. Furthermore, a potential challenge lies in determining the willingness of postpartum patients to continue acupuncture treatment until the study’s conclusion and to actively cooperate with the follow-up process.

This multicenter, randomized, sham-controlled trial will rigorously evaluate whether EA reduces the incidence of PUR and improves early bladder recovery; the results will help determine the clinical value of EA and may inform the use of non-pharmacologic strategies in postpartum rehabilitation.
