# Supplementary material for: Effect of electro-acupuncture on postpartum urinary retention: a protocol for multicenter, randomized and placebo-controlled trial
Source: Front Med (Lausanne). 2025 Nov 20;12:1698938. doi: 10.3389/fmed.2025.1698938 (PMC12677064; doi:10.3389/fmed.2025.1698938)
Supplement: Supplementary file 1 [file Data_Sheet_1.docx]

Supplementary Material

**Informed Consent Form for Clinical Trial Participants**

Dear Participant,

We would like to invite you to participate in a multicenter, randomized, parallel-controlled clinical trial investigating the impact of electroacupuncture intervention on postpartum urinary retention. The study aims to observe the effect of electroacupuncture on the incidence of postpartum urinary retention (PUR) and postpartum uterine contraction pain in patients.

Before you decide whether to participate in this study, please take the time to carefully read the following information. It will help you understand the purpose of the study, the procedures and duration involved, as well as the potential benefits, risks, and discomforts associated with participation. If you wish, you may discuss this with your family or friends, or seek clarification from your doctor to assist you in making an informed decision. The following is an introduction to the study:

**1. Overview and Objectives of the Study**

The purpose of this study is to investigate the short-term and long-term clinical effectiveness of electroacupuncture intervention in reducing the incidence of postpartum urinary retention (PUR) and to clarify the specific effects of acupoints.

The study will take place at four hospitals in China: the First Teaching Hospital of Tianjin University of Traditional Chinese Medicine, Tianjin Central Hospital of Gynecology Obstetrics, Tianjin Shuige Hospital and Second People's Hospital of Fengrun District, Tangshan city.The target enrollment is set at 660 patients.

Acupuncturists involved in this study possess years of experience and adhere strictly to the operational norms and quantifiable indicators for acupuncture. The team has independent ultrasound instruments and professional personnel to monitor urinary retention, assess postpartum urinary retention, uterine contraction pain, and maternal depression and anxiety. Vaginal ultrasound examinations will be uniformly conducted by dedicated personnel to minimize measurement bias.

This trial is supported by the national key laboratory of our hospital, providing the necessary instruments and equipment for the study. The team members have acquired the relevant clinical skills, ensuring reliable and objective conditions for the smooth progress of this research. The Ethics Committee of the First Affiliated Hospital of Tianjin University of Traditional Chinese Medicine has reviewed and approved the study in accordance with the principles of the Helsinki Declaration and medical ethics (Ethical Approval Number: TYLL2023[Z]027).

**2.Inclusion criteria:**

Patients meeting the following additional inclusion criteria may be included:

1. Primiparous women aged 20 to 45 years with singleton pregnancies planning for vaginal delivery.

2. Delivery at a gestational age of 37 to 42 weeks.

3. Being clearly conscious and cooperative.

4. Signing the informed consent.

**3.Exclusion criteria:**

Patients meeting any of the following conditions will be excluded:

1. Termination of pregnancy due to fetal malformation or stillbirth.

2. Severe central nervous system diseases, internal and surgical diseases, mental and psychological diseases, urogenital-related diseases, infectious diseases, and long-term drug treatment.

3. Severe coagulation disorders and a tendency towards spontaneous bleeding.

4. Skin damage at the acupuncture site.

5. Refusal to follow-up.

6. Refusal to sign the informed consent form.

**4. Research Procedures**

1. Before you are enrolled in the study, please sign this informed consent form.

2.Follow the steps outlined below during the study:

- The study will commence by determining whether you will receive treatment from Acupuncture Group ① or Sham Acupuncture Group ② based on a random number generated by a computer. Patients participating in this study have an equal chance (50%) of being assigned to either group, and neither you nor your doctor can predict or choose the group in advance.
- You should visit the hospital and accurately report any changes in your condition. The doctor will collect your medical history and examination results. Baseline measurements, including age, gestational weeks at delivery, height, pre-pregnancy weight, BMI, and method of conception, will be taken.
- Intervention Period: Acupuncture treatment will be administered within the first hour after delivery, on the first and second days postpartum, totaling three sessions. Bladder ultrasound monitoring will be conducted after the first postpartum voiding, 6 hours after delivery, and after each acupuncture treatment. We will also record your first voiding, uterine contraction pain, and postpartum bleeding. Edinburgh Postnatal Depression Scale assessment will be performed upon your discharge.
- After discharge, we will conduct outpatient follow-ups at 6-8 weeks to assess your voiding, pelvic floor function, and depression and anxiety. At one year postpartum, we will follow up on your voiding and pelvic floor function.

Please let us know if you have any questions or concerns before deciding to participate in the study.

**5. Responsibilities for Participation in the Study**

1.The study adheres to the principle of voluntary participation. Before the study begins, please sign the informed consent form voluntarily.

2.You are required to follow the scheduled follow-up appointments with the doctor. Your participation in these follow-ups is crucial, as the doctor will assess whether the research measures you are receiving are truly effective.

3.If you require any additional treatment during the study, please contact your doctor in advance.

Please feel free to ask any questions or seek clarification regarding your obligations in the study. Your commitment to these responsibilities is essential for the success of the research.

**6. Potential Benefits of Participation in the Study**

You and society may potentially benefit from this research. These benefits include the possibility of improvement in your medical condition and the potential development of a new treatment method that could be used for other patients with similar conditions.

During the study, you will receive excellent medical services. For the subsequent 2-day treatment period, you will receive free acupuncture treatment, ultrasound monitoring, as well as postpartum care and lifestyle guidance from experienced medical professionals tailored to your condition.

**7. Potential Risks and Discomfort/Inconvenience of Participation in the Study**

Any research study may entail certain risks, discomfort, or inconvenience. Acupuncture is a safe procedure, and there have been minimal reported side effects. Side effects may occur during or after treatment, such as transient and mild pain when the skin is pierced, minor bleeding upon needle withdrawal, bruising, nausea, dizziness, and fainting. These are generally short-lived and mild. Symptoms like nausea, dizziness, and fainting can be almost completely eliminated by adopting a supine position. If you experience any discomfort, please provide feedback so that appropriate guidance can be given.

If you experience any discomfort during the study, or if there are new changes in your condition or any unexpected situations, it is important to promptly inform your doctor. Your doctor will assess and provide appropriate medical care.

Moreover, there is a possibility that any treatment may prove ineffective, and the condition may continue to progress due to treatment ineffectiveness or the presence of other concurrent illnesses. During the study, if the doctor finds that the treatment measures taken in this research are ineffective, the study may be terminated, and alternative potentially effective treatment measures may be adopted.

You will need to attend follow-up visits at the hospital, complete certain questionnaires during the study, which may cause inconvenience or be troublesome for you.

**8. Handling of Research-Related Harm**

If you experience any harm related to the research during the clinical trial, the sponsor, the First Affiliated Hospital of Tianjin University of Traditional Chinese Medicine, will bear the costs of related treatment and provide corresponding financial compensation. This has been stipulated in the "Ethical Review Methods for Biomedical Research Involving Human Subjects" issued by the National Health Commission of China.

**9. Regarding Costs**

The Reproductive Medicine Department of the First Affiliated Hospital of Tianjin University of Traditional Chinese Medicine will cover the costs associated with acupuncture and ultrasound monitoring during your participation in this study.

However, if you require treatment and examinations for other concurrent illnesses, these will not be covered within the scope of the study and will incur separate charges.

**10. Willingness to Participate and Completion of the Study**

Your participation in the study is entirely voluntary. You have the right to refuse to participate or withdraw from the study at any time without affecting your relationship with your doctor. Your decision will not result in discrimination or unfair treatment, and it will not impact the provision of your regular medical services. When you decide not to continue with the study, we encourage you to inform your doctor promptly and complete any necessary examinations so that your doctor can provide appropriate advice or guidance based on your health status, which is beneficial for your health protection.

**11. Other Options Besides Participating in this Study**

You have the choice to participate or not in this study, and it will not have any adverse effects on your access to routine treatment. You are not obligated to choose participation in this study for the treatment of your condition. You also have the option to receive routine treatments provided by your doctor, such as catheterization, if deemed necessary.

**12. Circumstances Under Which You May Be Asked to Terminate Participation in the Study**

Your doctor or researcher, in consideration of your best interests, may decide to terminate your participation in this study at any time if any of the following circumstances arise:

1. Rescue measures are implemented due to postpartum hemorrhage, eclampsia, or other obstetric conditions;

2. Conversion from vaginal delivery to cesarean section

3. Infection is diagnosed during delivery;

4. An indwelling catheter is used and diuretics are administered due to other diseases within 6 hours after delivery;

5. A degree III laceration above the perineal fissure is diagnosed;

6. Due to other postpartum reasons, the number of treatment sessions received by patients is less than 3 times, which affects the analysis and judgment of the results;

7. The researchers can terminate the patients from the study at any stage if any events occur that may affect the safety of patients in the study.

If you choose to withdraw from the study before completion, for safety considerations, we may collect samples for relevant safety checks. If, after your withdrawal, new information emerges that is relevant to your health and rights, we may contact you again to discuss this information. Your safety and well-being remain a priority throughout the study and beyond.

**13. Confidentiality of Personal Information**

Your medical records (CRF, laboratory reports, etc.) will be securely stored at the hospital, and the doctor will document the results of laboratory tests in your medical records. Ethical committee and research management personnel may be allowed to access your original medical records for inspection without violating confidentiality principles and relevant regulations. Any public reports related to the results of this study will not disclose your personal identity. We will make every effort, within the limits permitted by law, to protect the privacy of your personal medical information.

Apart from this study, your medical records may potentially be used in future research. You have the option to declare your refusal for the use of your medical records in any other research besides the current study.

**14. How to Obtain More Information?**

You can ask any questions about this study at any time. Your doctor will provide you with their contact information to address any concerns or questions you may have. If there is any important new information during the course of the study that may affect your willingness to continue participating, your doctor will inform you promptly.

The study has been reviewed and approved by the Ethics Committee. If you have any questions related to your rights and interests or if you wish to report any difficulties, dissatisfaction, or concerns encountered during your participation in the study, or if you want to provide opinions and suggestions related to the study, please contact the Ethics Committee Office at (022) 27986258 or via email at yfyiec@163.com.

**15. What to Do Now?**

The decision to participate in this study is entirely yours. You may discuss it with your family or friends before making a decision.

Before making a decision to participate, please ask your doctor any questions you may have until you have a complete understanding of the study.

Thank you for reading the information provided. If you decide to participate in this study, please inform your doctor or research assistant, and they will assist you with all the necessary arrangements related to the study.

Please keep a copy of this information for your reference.

**Informed Consent Form · Consent Signature Page**

I, ________________________ (Participant's Name), have thoroughly read the Informed Consent Form for the clinical research project titled "The Impact of Electroacupuncture Intervention on Postpartum Urinary Retention – A Multicenter, Randomized, Parallel-Controlled Clinical Trial."

I have gained a comprehensive understanding of the study's purpose, procedures, risks, and benefits. Based on a clear comprehension of the research, I voluntarily choose to participate in this study.

I understand any potential risks and discomfort that may arise during the study and agree to participate in accordance with the research plan and the advice of the medical team. I acknowledge my right to voluntarily withdraw from the study at any time without affecting my regular medical care at the hospital.

I agree that, for safety considerations, the research team may collect relevant samples for safety checks after my withdrawal from the study. I also consent to being contacted again by the research team if new information arises that is relevant to my health and rights.

I understand that my personal medical information will be kept confidential, and I consent to the research team and the ethics committee accessing my medical records within the bounds of relevant regulations and confidentiality principles.

I have raised all the questions I am concerned about to the research team and have received satisfactory answers.

Participant's Signature: ________________________

Date: ________________________

(Please ensure that you fully understand and agree to participate in the study before signing.)

I confirm that I have explained the details of this trial to the participant, including their rights, potential benefits, and risks. I have provided them with a signed copy of the informed consent form.

Doctor's Signature: ________________________

Date: ________________________
